# A comparison of children’s diet and movement behaviour patterns derived from three unsupervised multivariate methods

**DOI:** 10.1371/journal.pone.0255203

**Published:** 2021-07-27

**Authors:** Ninoshka J. D’Souza, Katherine Downing, Gavin Abbott, Liliana Orellana, Sandrine Lioret, Karen J. Campbell, Kylie D. Hesketh

**Affiliations:** 1 Institute of Physical Activity and Nutrition, School of Exercise and Nutrition Sciences, Deakin University, Geelong, Victoria, Australia; 2 Biostatistics Unit, Deakin University, Geelong, Victoria, Australia; 3 Research Center in Epidemiology and Biostatistics (CRESS), INSERM, INRAE, Université de Paris, Paris, France; Curtin University, AUSTRALIA

## Abstract

**Background:**

Behavioural patterns are typically derived using unsupervised multivariate methods such as principal component analysis (PCA), latent profile analysis (LPA) and cluster analysis (CA). Comparability and congruence between the patterns derived from these methods has not been previously investigated, thus it’s unclear whether patterns from studies using different methods are directly comparable. This study aimed to compare behavioural patterns derived across diet, physical activity, sedentary behaviour and sleep domains, using PCA, LPA and CA in a single dataset.

**Methods:**

Parent-report and accelerometry data from the second wave (2011/12; child age 6-8y, n = 432) of the HAPPY cohort study (Melbourne, Australia) were used to derive behavioural patterns using PCA, LPA and CA. Standardized variables assessing diet (intake of fruit, vegetable, sweet, and savoury discretionary items), physical activity (moderate- to vigorous-intensity physical activity [MVPA] from accelerometry, organised sport duration and outdoor playtime from parent report), sedentary behaviour (sedentary time from accelerometry, screen time, videogames and quiet playtime from parent report) and sleep (daily sleep duration) were included in the analyses. For each method, commonly used criteria for pattern retention were applied.

**Results:**

PCA produced four patterns whereas LPA and CA each generated three patterns. Despite the number and characterisation of the behavioural patterns derived being non-identical, each method identified a healthy, unhealthy and a mixed pattern. Three common underlying themes emerged across the methods for each type of pattern: (i) High fruit and vegetable intake and high outdoor play (“healthy”); (ii) poor diet (either low fruit and vegetable intake or high discretionary food intake) and high sedentary behaviour (“unhealthy”); and (iii) high MVPA, poor diet (as defined above) and low sedentary time (“mixed”).

**Conclusion:**

Within this sample, despite differences in the number of patterns derived by each method, a good degree of concordance across pattern characteristics was seen between the methods. Differences between patterns could be attributable to the underpinning statistical technique of each method. Therefore, acknowledging the differences between the methods and ensuring thorough documentation of the pattern derivation analyses is essential to inform comparison of patterns derived through a range of approaches across studies.

## Introduction

For children, diet and time spent in physical activity, sedentary behaviour, and sleep are key behaviours implicated in disease development [1–3]. Previous work has scrutinised the role of these behaviours individually; however, research regarding their synergistic or combined effects is an emerging area [3–5]. There is evidence an integrated approach that evaluates behavioural patterns will improve our understanding of complex health outcomes in children [3, 6–8].

Behavioural patterns are typically derived using unsupervised learning, a type of algorithm that does not involve a priori labelling or classification of the responses according to external criteria/guidelines [9, 10], but instead are data driven. The most common unsupervised learning methods used in the nutrition and physical activity field include principal component analysis (PCA), cluster analysis (CA), and latent class/profile analysis (LCA/LPA, for categorical and continuous input data respectively) [10]. A key distinction between the methods is their focus for grouping data (based on variables or individuals) and the resultant output type. PCA focuses on *variables*, identifying groups of highly correlated variables [11] using the covariance or correlation matrix [12] and transforming them into a smaller number of new, linearly uncorrelated variables known as principal components [13]. Each individual will have a ‘score’ for each of these principal components. Conversely, both CA and LCA/LPA focus on *individuals*, finding groups of individuals with similar characteristics [14] and assigning them into mutually exclusive clusters [13]. Individuals in different clusters have different patterns of the input variables. LCA/LPA also focus on individuals but assume that in the population there are “latent classes” or sub-types of individuals that have similar values of the input variables. Class membership of individuals is unknown but can be inferred from the input variables [15, 16]. All three methods can identify behaviours that are likely to co-occur in the same individual [10, 17]. Key differences across methods are further highlighted in Table 1.

**Table 1:** Brief summary of pattern derivation methods.

Methods	Principal component analysis	Cluster analysis	Latent profile analysis
Description	Data reduction technique that summarizes input variables into components which are linear combinations of the input variables.	Classification method that summarises input variables into clusters which are homogenous (comprising individuals with similar characteristics).	Classification method that summarizes input variables into latent profiles, where members are assigned probabilities of belonging to a profile.
Number of patterns	Initially equal to the number of input variables but number of patterns retained is based on criterion decided by the researcher (e.g., Horn’s parallel analysis or eigenvalue cut-offs).	A number of criteria exist to decide clusters to be retained, which include the Calinksi-Harabasz statistic for K-means clustering.	Determined using statistical model-based criteria which include the BIC and aLMR test (used in the present study). Other common criteria include the AIC and the BLRT test.
Type of output variable	Continuous (component scores).	Categorical (cluster membership).	Categorical (after allocating each individual to their most probable profile membership).

Abbreviations: BIC; Bayesian information criteria, aLMR; adjusted Lo-Mendel-Rubin test, AIC; Akaike information criteria, BLRT, bootstrap likelihood ratio test.

A limited number of studies have compared behavioural patterns derived from multiple unsupervised learning methods within a single dataset. Two studies in adults compared dietary patterns, one [9] using PCA and CA and the other [18] using CA, PCA and index analysis (an investigator-driven method). They observed some similarity between pattern characteristics, despite different numbers of patterns being derived from the methods. They suggested that some direct comparisons are possible, however, the research question/objective should guide the choice of method for analyses. Based on the number of patterns obtained, they suggested those methods to be better if they provided more information, i.e., “patterns” from the sample, however, these conclusions are not generalizable. In another study [14], CA and LPA were used to derive different numbers of patterns of physical activity and sedentary behaviour in adolescents. The authors concluded that based on statistical technique, LPA overcomes certain limitations of CA and, therefore, may be a more reliable choice. Previous studies have been limited to patterns within behaviour domains; either diet or movement (physical activity/sedentary behaviour) [19]. To our knowledge, no studies have compared patterns derived from PCA, CA and LCA/LPA across lifestyle behaviours—diet, physical activity, sedentary behaviour, and sleep.

The various methods can produce different patterns (type/number) when applied to the same dataset [9, 14]. Differing results are possible even for the same approach due to the multiple decisions involved when deriving patterns, such as the definition/grouping/treatment of the initial variables, selection of the statistical criteria and/or subjective decisions made by the investigator [13, 14]. Lastly, interpretation of the output also influences the final patterns retained. Despite different solutions (i.e., number of and composition of patterns) being possible from different methods, under the assumption that a small number of behavioural patterns exist within the target population, it is of interest to explore if these methods identify at least a core set of behavioural patterns that are comparable. Previous studies have focused on identifying most suitable/ideal/common patterns in their study populations and only a few [14, 20] have discussed discrepancies that can arise from using different methods.

This paper aimed to investigate congruence between behavioural patterns across diet, physical activity, sedentary behaviour and sleep domains, derived using PCA, LPA and CA. This was assessed in a dataset of 6- to 8-year-old children.

## Methods

### Data source

Data were drawn from the Healthy Active Preschool and Primary Years (HAPPY) cohort. Details of the study are described in detail elsewhere [21]. In brief, the baseline sample (2008/09) included 1002 parents and their children aged 3-5y, recruited through preschools and childcare centres in Melbourne, Australia. Children were followed up at multiple time points (76% of the initial sample [n = 776] provided consent to be followed up). This study draws on data from the second wave in 2011/12 when children were aged 6-8y. In the second wave, 567 children participated, with 432 providing complete data who were included in this study. HAPPY was granted ethical approval from the Deakin University Human Research Ethics Committee (EC 291–2007), the Department of Education and Early Childhood Development (2011_001008) and the Catholic Education Office (1714). At each time point, parents provided written informed consent for themselves and their child to participate.

### Measures and data management

#### Diet

Parents reported child dietary intake using a previously validated 15-item food frequency questionnaire [22]. Included items were those showing acceptable reliability (ICC > 0.6) and validity. Frequency of a range of discretionary foods eaten in the previous week was recorded using a 7-point Likert scale (0–6 or more times). Six sweet (spreads [peanut butter or Nutella], pre-sugared cereals, bakery items, lollies and snack bars, chocolate, ice-cream) and seven savoury (potato crisps or savoury biscuits, cheese and cheese spreads, pies and sausage rolls, pizza, hot chips or French fries, hot dogs and processed meats and takeaway foods) discretionary food items were summed and divided by seven to obtain daily intakes. The frequency of fruit and vegetables consumed in the past 24 hours (representing daily intake) were recorded using a 6-point Likert scale (0–5 or more times).

#### Physical activity

Physical activity was objectively measured using ActiGraph GT1M uniaxial accelerometers (Pensacola, FL, USA), worn for eight consecutive days. Accelerometers were worn at the hip, during waking hours and removed for water-based activities. Non-wear time > 20 minutes was classified as zero counts. Accelerometer data were considered valid if data were recorded for at least eight hours a day for four or more days, inclusive of at least one weekend day [23]. Accelerometer data were classified as moderate- to vigorous-intensity physical activity (MVPA) for counts >2296/min [23]. Additionally, parents reported information on total weekly duration of different organised sports their child engaged in, using a reliable survey [24]. These included swimming, gymnastics, dance, football, soccer, netball, basketball, and cricket, and also accounted for other sports not included in the list with an open choice ‘other’ category. Total weekly organised sport duration was obtained by summing the total weekly duration for each sport. This was divided by seven to derive daily equivalents. Parents also reported the time their child spent in outdoor play on a typical weekday and weekend day. This was weighted to obtain an average daily time in minutes, i.e., the sum of daily weekday time and daily weekend time averaged over five and two days respectively, divided by seven. Test retest reliability was acceptable for organised sport (ICC = 0.74) and outdoor play (ICC = 0.44) [24].

#### Sedentary behaviour

Sedentary behaviour was assessed by accelerometry (as described above) and parent-report. Accelerometer data of <100 counts per minute was classified as sedentary time [25] and reported as minutes per day. Parents reported the total number of hours their child usually spent in leisure-time sedentary behaviours during the week (Monday to Friday) and on weekends (Saturday and Sunday). Evidence suggests that children’s screen time can be both active (e.g., videogames) and passive (e.g., television viewing) in terms of child engagement, and different modes can have differential effects on health [26]. These behaviours were therefore treated separately and categorized into screen time (television viewing and computer use excluding games), video game use (including computer games and handheld electronic games) and quiet playtime. When assessed for test-retest reliability these items showed low to moderate acceptability (ICC = 0.10 [quiet play], ICC = 0.44 [screen time] and ICC = 0.68 [video game use]) [21]. Daily duration for each of these categories was calculated by summing the weekday and weekend time duration and dividing by seven. The low reliability of these items was inferred to be due to true day-to-day variability in these behaviours assessed two weeks apart, rather than responses being inaccurate and unreliable themselves [24].

#### Sleep

Parents reported their child’s usual nightly sleep duration in hours and minutes per night. This item showed good test retest reliability (ICC = 0.82) [24].

### Data analysis

Stata 15.0 (StataCorp, Texas, USA) was used for CA and PCA and Mplus 8.0 for LPA. All analyses included four dietary (intake of fruit, vegetables, sweet and savoury discretionary items), three physical activity (MVPA from accelerometry, organised sport duration, and outdoor playtime), four sedentary behaviour (sedentary time from accelerometry, screen time, videogames, and quiet playtime) and one sleep (daily sleep duration) variable. Using the residuals obtained from regressing accelerometry data on wear time, accelerometer variables (both MVPA and sedentary time) were adjusted for daily wear time (26). Input variables were converted to standardised scores (mean = 0, SD = 1) as they were measured using different scales.

#### Cluster analysis

Non-hierarchical K-means CA was performed. Since this method requires pre-specification of the number of clusters to be generated, a range of cluster solutions (2–10 clusters) were derived. The Calinski-Harabasz statistic, which measures improvement in some measures of fit between models, was then used to determine the optimal number of clusters [27]. Higher values for this statistic indicate better fit when comparing different number of clusters [27]. Post-assessment of the values from the Calinski-Harabasz statistic, the size of the clusters across the different cluster solutions and their interpretability and meaningfulness [13, 28] were considered to determine the final number of clusters. The Calinski-Harabasz statistic indicated a 2-cluster model as optimal; however, upon assessment of cluster size, it was found that two large and distinct clusters from the 3-cluster model were combined in this solution, with the remaining cluster unchanged across the 2- and 3- cluster models. Upon inspection, the 3-cluster model was considered more informative and hence selected.

#### Latent profile analysis

Since LPA requires pre-specification of the number of profiles (homogeneous subgroups), multiple profile models (2–10 profiles) were derived and then compared using two recommended model fit criteria; the Bayesian information criteria (BIC) and the adjusted Lo-Mendel-Rubin (aLMR) test. Models with lower values for the BIC indicate better fit [29]. The aLMR test compares fit between neighbouring profile models [16] and when the test does not reject the null-hypothesis the model with smaller number of classes is preferred [29]. BIC and aLMR model estimation criteria were inconsistent, suggesting 9-profile and 3-profile models, respectively, as optimal. Upon inspection, the 3-profile model was more interpretable and logical, hence selected. A general cut point of ±0.2 was used to interpret behaviours that were high/low in the patterns derived from LPA and CA.

#### Principal component analysis

The number of principal components to retain was determined using Horn’s parallel analysis, where eigenvalues are compared to the mean of eigenvalues estimated from a Monte-Carlo simulated matrix of the same size. Horn’s parallel analysis accounts for the sample bias in estimating the eigenvectors and eigenvalues and provides a more accurate and advanced approach than the Kaiser criterion which keeps principal components with eigenvalue greater than one [13, 30]. Varimax rotation was performed to obtain more interpretable component loadings and subsequently more contrasted patterns. When interpreting the derived principal components coefficients (loadings) all input variables were considered.

As these three statistical methods are potentially sensitive to outliers, the same analyses (results not shown) were conducted excluding outliers. Only minor differences (e.g., slightly different coefficients) in the pattern solutions were observed therefore results are reported on the full data set to maximise sample size and generalisability of results.

## Results

**Fig 1:**
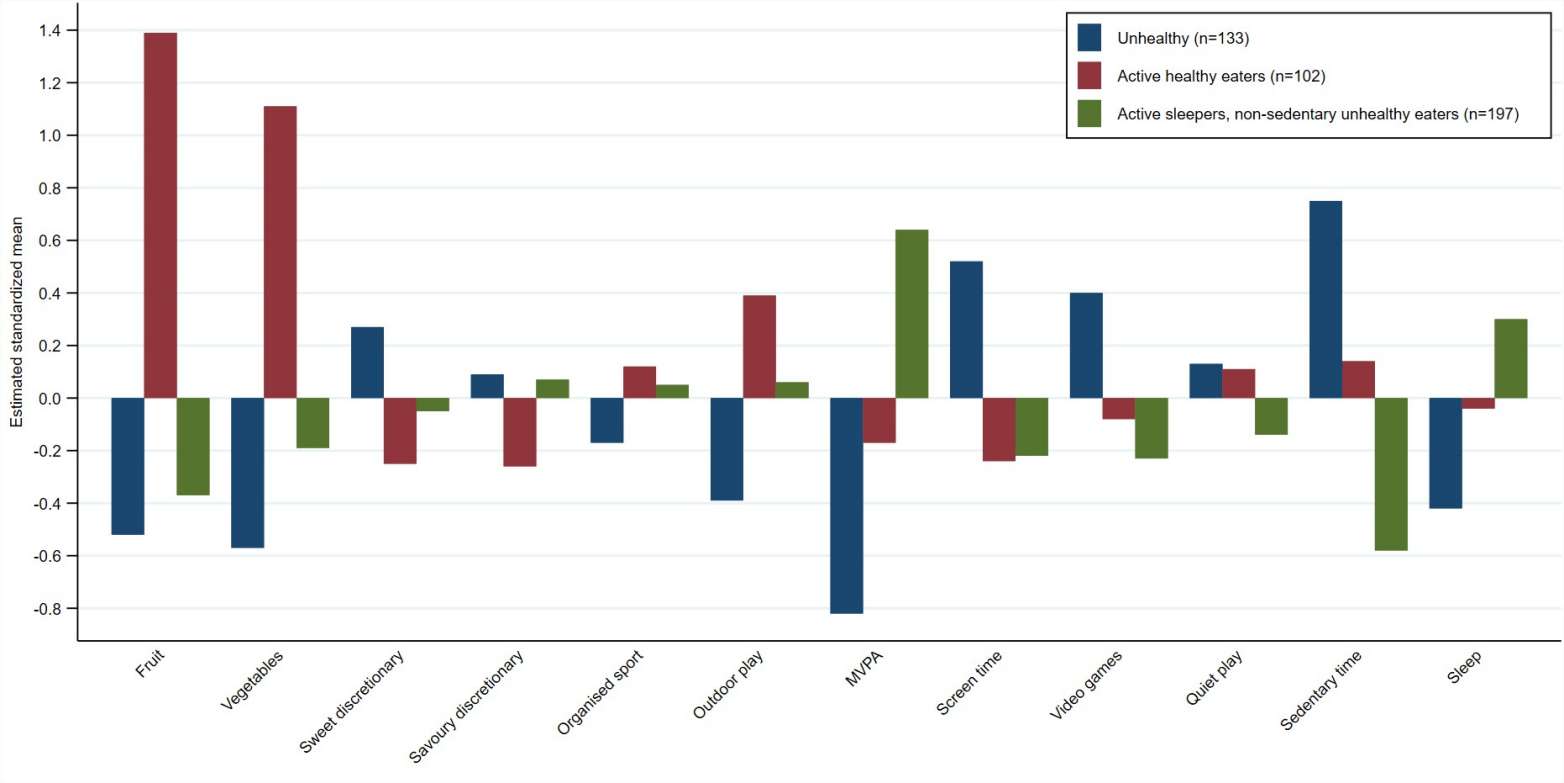
Average value of the standardized input variables for each behavioural pattern derived using K-means cluster analysis. Footnote: n; number of children in each pattern.

**Fig 2:**
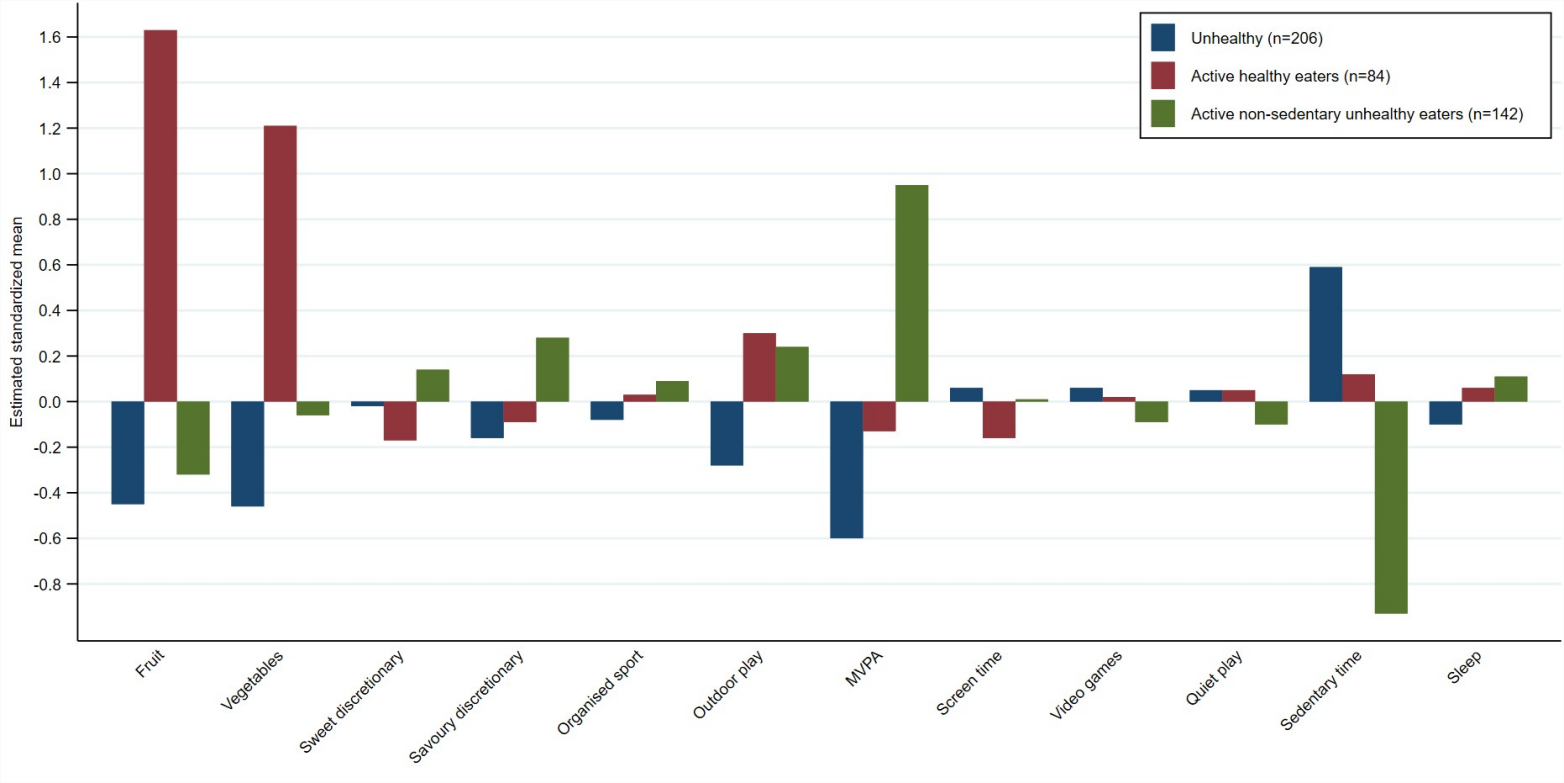
Average value of the standardized input variables for the behavioural patterns derived using latent profile analysis. Footnote: n; number of children in each pattern.

**Fig 3:**
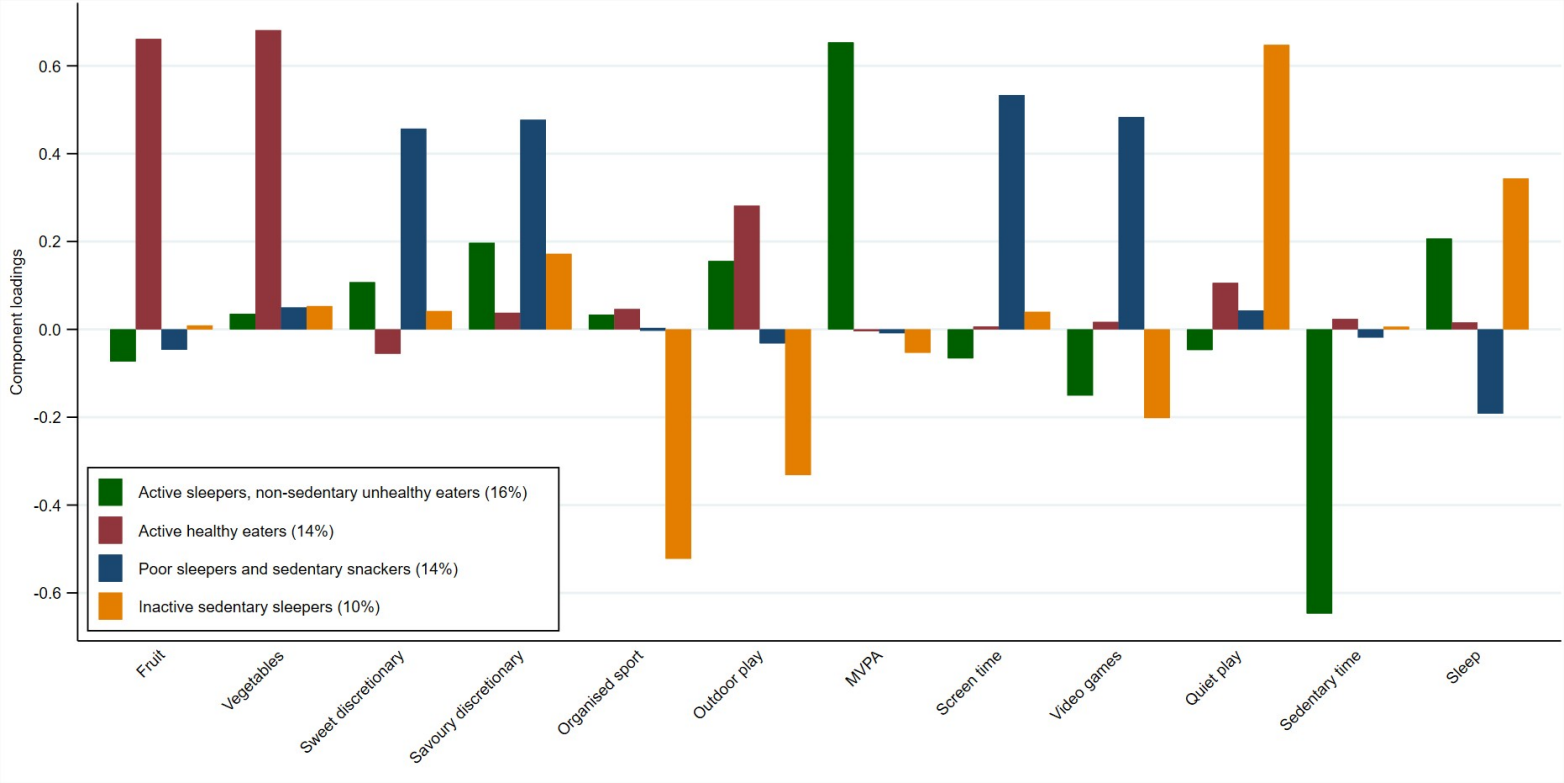
Component loadings for each behavioural pattern derived using principal component analysis. Footnote: The percentage after each pattern label is the percent of the total data variance explained by that pattern.

Participants (n = 432) with complete survey and accelerometer data were included in all analyses. Sample characteristics are presented in Table 2. Four distinct patterns were derived from PCA, and three from CA and LPA. Despite these differences, each method identified a pattern that could be described as ‘healthy’; that is, reflective of a healthy diet, high physical activity, low sedentary behaviour, and high sleep duration. Patterns with characteristics contrary to healthy behaviours were also found using each of the three methods and were described as ‘unhealthy’. Lastly, patterns containing a mixture of healthy and unhealthy behaviours were identified using all three methods and were described as ‘mixed’. Across LPA and CA, a large proportion of children (76%) were classified into the same pattern type (either unhealthy/unhealthy or mixed) whilst the remaining children were classified inconsistently. Patterns derived from each method are presented in Figs 1–3 and mean z-scores/component loadings are presented in S1 Table.

**Table 2:** Characteristics of the sample included in the analysis (n = 432).

Characteristic	Mean (SD)	Median (IQR)	Range
Child age (years)	7.6 (0.7)	7.6 (7.0–8.2)	5.4–9.1
Sex [n (%)]		-	-
Male	244 (56.5)		
Female	188 (43.5)		
Diet			
Fruit intake (times/day)	2.3 (1.3)	2.0 (1.0–3.0)	– 5.0
Vegetable intake (times/day)	2.9 (1.3)	3.0 (2.0–4.0)	0.0–5.0
Sweet discretionary food intake (times/day)	1.5 (0.7)	1.4 (1.0–2.0)	0.0–3.7
Savoury discretionary food intake (times/day)	1.2 (0.5)	1.1 (0.8–1.6)	0.0–2.6
Physical activity			
Organised sport (mins/day)	24.2 (20.0)	21.4 (12.9–32.1)	0.0–197.1
Outdoor play (mins/day)	143.0 (77.5)	132.1 (94.3–180/0)	6.4–617.1
MVPA (mins/day)^a^	106.6 (28.0)	104.7 (86.1–124.1)	42.9–190.0
Sedentary behaviour			
Screen time (mins/day)	93.0 (57.9)	77.1 (47.1–128.6)	– 321.4
Videogames (mins/day)	23.2 (30.7)	12.9 (0.0–34.3)	– 214.3
Quiet play time (mins/day)	52.5 (39.7)	42.9 (25.7–68.6)	0.0–342.9
Sedentary time (mins/day)^a^	(41.7)	362.3 (332.5–390.4)	247.9–487.7
Sleep (mins/day)	623.3 (47.4)	630 (600.0–660.0)	450.0–720.0

Abbreviations: mins–minutes, IQR–interquartile range, MVPA–moderate- to vigorous-intensity physical activity, PA–physical activity, SB–sedentary behaviour, SD–standard deviation.

^a^ Mean accelerometer wear time was 703 minutes per day.

Cluster Analysis using K-means identified three clusters (see Fig 1). Cluster 1 (n = 133), labelled ‘**unhealthy**’, was characterised by lowest fruit and vegetable intake, lowest overall physical activity (organized sport, outdoor play duration and MVPA), lowest sleep, highest sweet discretionary food intake and highest sedentary behaviour (screen, videogame, and sedentary time). Cluster 2 (n = 102), labelled ‘**active healthy eaters**’ (healthy), was characterised by highest fruit and vegetable intake, highest outdoor play time, lowest discretionary food intake and lowest screen time. Cluster 3 (n = 197), labelled ‘**active sleepers, non-sedentary unhealthy eaters**’ (mixed), was characterised by highest MVPA levels, highest sleep duration, low fruit intake and lowest videogame and sedentary time.

For LPA (see Fig 2), Profile 1 (n = 206) was labelled ‘**unhealthy**’ and comprised of lowest fruit and vegetable intake, lowest outdoor play time and MVPA, and highest sedentary time. Profile 2 (n = 84), labelled ‘**active healthy eaters**’ (healthy), was characterised by highest fruit and vegetable intake and highest outdoor play time. Profile 3 (n = 142), ‘**active non-sedentary unhealthy eaters**’ (mixed), was characterised by highest savoury discretionary food intake, highest MVPA, high outdoor play time, low fruit intake and lowest sedentary time.

Four principal components were retained according to Horn’s parallel analysis, which explained 54% of the total variance. These four patterns (see Fig 3) were labelled: ‘**active sleepers, non-sedentary unhealthy eaters**’ (mixed), (explained 16% of variance), characterised by intake of savoury discretionary food items, high MVPA, adequate sleep duration and low sedentary time; ‘**active healthy eaters**’ (healthy), (explained 14% of variance), comprising high fruit and vegetable consumption and outdoor play time; ‘**poor sleepers and sedentary snackers**’ (unhealthy), (explained 14% of variance), comprised of high discretionary food intake, high screen and videogame time, and lowest sleep duration; and ‘**inactive sedentary sleepers**’ (mixed), (explained 10% of variance), characterised by high quiet play time and sleep, and low organised sport and outdoor play time.

A three-component PCA model with varimax rotation (explaining 44% of the total variance) was additionally derived to allow direct comparison with the three patterns obtained from CA and LPA. The patterns identified were the same as the first three patterns from the four component PCA solution (results not presented).

## Discussion

To our knowledge, this was the first study to compare patterns derived from four behavioural domains (diet, physical activity, sedentary behaviour, and sleep) in children using three different analytic methods, PCA, LPA and CA. Patterns identified by different methods were not identical; however, some similar underlying themes emerged between the three methods indicating that such methods can identify ‘a core set’ of underlying patterns. The number of patterns identified were not uniform across all methods, however, each method identified a healthy, unhealthy, and mixed behavioural pattern. These core patterns were on the whole comparable to those reported in previous reviews [2, 19, 31] of behavioural patterns in children, where healthy, unhealthy, and mixed patterns have been identified despite variation in the specifics of such patterns and in the underlying behaviours analysed in different studies. It is, however, apparent that clear reporting of analytical techniques used and decisions made is important to enable assessment of comparability across studies given such decisions do influence the finer detail of the patterns derived.

Patterns derived across methods in the present study were comparable to previous studies. Healthy patterns characterised by outdoor play and healthy eating have similarly been reported in three other studies [32–35], all using PCA. Similar unhealthy patterns comprising an unhealthy diet and high sedentary behaviour were reported in nine studies, five using CA [28, 36–39] and four using PCA [32, 34, 35, 40]. The mixed pattern, comprising high MVPA, high discretionary food intake and low sedentary time, was somewhat similar to the patterns derived by Seghers et al. [41]. However, the other mixed pattern from our study, characterised by high quiet play time, sleep and low organised sport and outdoor play time is unique. Few previous studies have included objectively assessed physical activity data in their analyses which may explain why this pattern has not been reported previously. While similar patterns can be identified in the literature, they have not been specifically observed within a single previous study. The discrepancies in pattern solutions possible can be attributed not only to the specific input variables used to assess behaviours in each study or to cultural differences between the study populations, but also to specific statistical criteria chosen and subjective decisions made when applying these methods, and also due to a broader range of behaviours included in this study. Future studies should consider detailed documentation of their methodological approaches to enable better comparisons of patterns across studies. Standardised reporting for these methodological approaches is also warranted.

When a three-pattern solution was compared across all methods, high concordance was observed with all methods identifying a healthy, unhealthy and a mixed pattern. As expected, patterns derived from LPA and CA were more similar than PCA. Additionally, most children were classified consistently into the same pattern type across LPA and CA, however, these comparisons are not as straightforward with PCA as children have scores for all patterns. PCA is a dimension-reduction method that looks for correlated variables, whereas LPA and CA look for similarities between individuals [11, 13]. One quarter of the sample was classified into different pattern types by CA and LPA; this could be due to the approach used by each method to assign pattern membership. CA uses a distance-based similarity index, whereas LPA estimates individual probabilities for each pattern derived and then assigns an individual into their most probable pattern. Despite underlying differences of how these methods derive patterns, the three approaches identified patterns that were promotive (healthy) and demotive (unhealthy, mixed) of health. This suggests there is reasonable overlap in the patterns derived from these different methods, with the choice of method more dependent on the preferred output type (categorical/continuous) or statistical technique (probabilistic/geometrical etc.). Nonetheless, given that the patterns derived were not identical (as expected, given the differing underlying algorithms used for pattern derivation by the different methods) and a quarter of the sample were classified into different pattern types inconsistently across two methods (CA and LPA), direct comparisons of patterns from studies using different methods must be interpreted with caution. These findings suggest that the method chosen to derive patterns may influence conclusions about associations of behavioural patterns with health outcomes. Studies assessing comparisons of associations with health outcomes using patterns derived from different methods are warranted to confirm this.

Each method has inherent strengths and limitations and a set of analyst decisions involved. Although within each method there is no absolute correct model, given the true underlying population patterns are unknown, it is essential authors justify decisions made and the final model chosen. For example, in our case, choosing the three instead of the four-pattern PCA model would not be incorrect, however, it explains a smaller proportion of the data total variance (44% versus 54%) and hence was deemed less ideal. Horn’s parallel analysis provides an objective way to determine the number of principal components [30] but has not been frequently used or reported in previous studies using PCA, with the more inaccurate/biased Kaiser criteria typically used. Horn’s parallel analysis generally outperforms approaches such as the Kaiser criteria, yet due to it being more computationally intensive, simpler methods such as the Kaiser criteria had much higher adoption [42]. This practice has largely persisted despite today’s computers being adequate for Horn’s parallel analyses in most cases. Standard implementation of this technique across widely used statistical packages is also lacking [42]. The use of Horn’s parallel analysis may change the number of components to be selected and thus may be useful for future investigators to consider, with the potential to provide greater consistency across studies. PCA provides scores for each sample member for all patterns derived and is useful for those studies requiring continuous variables for subsequent analysis [11]. Patterns derived from CA can vary widely due to the vast number of possibilities for the clustering algorithm and the parameter settings. Additionally, the heterogeneity of cluster members is ignored once clusters have been defined, as members within a cluster are considered homogenous. This may be disadvantageous as the internal variation may be extremely large, particularly when the number of clusters is small [18]. Large internal variation can introduce classification errors, thus impairing statistical power in subsequent analyses. Evaluating the best model fit for LPA was challenging as the preferred number of profiles based on the two criteria were not concordant. Although the BIC has been shown to outperform other model estimation criteria [16, 43], the model it suggested was not logical or interpretable. In comparison, the aLMR test provided a clear optimal number of profiles which were most interpretable in this study. Latent profile analysis is superior to cluster analysis in its ability to (a) provide individual probabilities for the different profiles identified, (b) account for missing data and (c) include health outcomes while deriving patterns, all while considering the probability of misclassification [20]. Although PCA produces variables/components being a data reduction method, and CA and LPA are classification methods which group individuals, they are nonetheless somewhat comparable as they indirectly provide similar information; variables from PCA inform us of behavioural patterns expressed by individuals, whereas groups of individuals from CA and LPA express behavioural patterns. It is important to consider the strengths and limitations of each method within the context of the individual study and the research question being asked to decide on the most appropriate method.

The study is not without some limitations and strengths. This study did not investigate an exhaustive list of statistical methods to derive patterns, rather opting for the most common methods reported in the literature. Within each method, several pattern solutions are possible and analyst decisions influence outcomes. Rather than investigating all possible alternatives, we utilised the most common approaches reported in the literature within each method. Limitations of the individual methods are described above, however, there are also some limitations common across all methods. These pattern derivation methods are sensitive to outliers and can be influenced by the distribution of the input variables [11], however, the fairly large sample in this study provided some protection against this. Future studies could also benefit from a larger sample size, as this study sample was limited by the availability of complete data for all 12 behaviours included in the pattern derivation analyses.

The final patterns obtained are dependent on decisions taken, given there is no single approach to derive patterns. Model estimation criteria may not always provide logical patterns (as seen for LPA in this study), therefore, future studies should consider balancing objective (model estimation criteria) and subjective decision making. This will ensure the patterns defined are valid based on the estimation criteria but also are pragmatic and logical using sound subjective decision-making. This overcomes some limitations of using a-priori methods which are predominantly analyst decision driven involving pre-classification of behaviours, thereby affecting final patterns derived. Most importantly, thorough documentation of all decisions taken will be crucial to assess comparability of their findings with other studies.

We acknowledge potential recall and social desirability bias introduced by using survey data [44, 45], in addition to the reliability of some items in the survey being low. Parent-report data can limit measurement precision (explaining some of the discrepancies across methods) and also lead to over/under estimations of healthy/unhealthy behaviours respectively, due to social desirability bias, thereby potentially affecting the final patterns derived and subsequent associations. Given our primary aim was to compare patterns from different techniques in a single dataset, and since most previous studies used subjective measures, this is less concerning. The use of subjective and objective measures provides some balance between accuracy and context-rich information for some behaviours (physical activity and sedentary behaviour). Although objective measures help refine the accuracy of patterns obtained, using them alone cannot distinguish different activity types (context-based) within behavioural domains. The inclusion of quiet play time (in the sedentary behaviour domain) and sleep duration is novel, as these variables have not been frequently included in previous pattern derivation analyses. The diversity of the variables included across the behavioural domains to derive patterns is an additional strength of this study as it helps to better understand the interplay of these behaviours, ultimately valuable in health prevention efforts.

In summary, our findings indicate that while there are some similarities in patterns identified using different methods, there are also notable differences. The similarity in pattern characteristics across methods may help provide some confidence in the underlying patterns prevalent in a given dataset. However, the differences observed across patterns have potential to influence subsequent analyses of associations with various outcomes. Such differences are yet to be investigated, but they suggest that the choice of method may influence associations with health outcomes and might explain the discrepancy in findings reported across studies. Thus, comparison of findings across studies employing different pattern derivation methods should be done with caution. Whilst the goal of this study was not to recommend one method over the other, as each has strengths and weaknesses, future researchers should consider the choice of method based on their study objectives and subsequent analyses.

## Conclusion

Typically, health behaviours (e.g., diet, physical activity, sedentary behaviour, and sleep) are considered individually as predictors of health. However, these behaviours do not occur in isolation and are likely patterned as shown in the present study. Consequently, many studies are now deriving and reporting patterns of behaviour to help describe their synergistic influence. Multivariate methods such as PCA, LPA and CA are useful in identifying behavioural patterns in a given dataset. In this study, each of the three methods identified a core set of underlying patterns characteristic of a healthy, unhealthy and mixed pattern. The similarities provide greater confidence that the patterns are present in the target population. However, the patterns identified by the different methods were not identical. The differences can be attributable to the algorithms underpinning each method and highlight the importance of documenting not only the methods used, but the objective and subjective decisions taken in the analytic process. Overall, comparison of patterns at a broad level using different methods is possible. However, when comparing the finer details of pattern characteristics across studies utilising different methods, it is important to be mindful that the differences may be an artefact of the statistical techniques used rather than reflective of true differences in the samples.

## Supporting information

S1 Table Pattern characteristics for each method–component loadings (for PCA) and mean z-scores (for LPA/CA). Abbreviations: CA–cluster analysis, LPA–latent profile analysis, mins–minutes, MVPA–moderate- to vigorous-intensity physical activity, PA–physical activity, PCA–principal component analysis, SB–sedentary behaviour.
(DOCX)
