# Supplementary material for: A comparison of children’s diet and movement behaviour patterns derived from three unsupervised multivariate methods
Source: PLoS One. 2021 Jul 27;16(7):e0255203. doi: 10.1371/journal.pone.0255203 (PMC8315509; doi:10.1371/journal.pone.0255203)
Supplement: S1 Table — Abbreviations: CA–cluster analysis, LPA–latent profile analysis, mins–minutes, MVPA–moderate- to vigorous-intensity physical activity, PA–physical activity, PCA–principal component analysis, SB–sedentary behaviour. (DOCX) [file pone.0255203.s001.docx]

S1 Table: Pattern characteristics for each method - Factor loadings (for PCA) and mean z-scores (for LPA/CA)

|  | Variables | CA | | | LPA | | | PCA | | | |
| --- | --- | --- | --- | --- | --- | --- | --- | --- | --- | --- | --- |
|  |  | 1 | 2 | 3 | 1 | 2 | 3 | 1 | 2 | 3 | 4 |
| Diet | Fruit intake (times/day) | -0.52 | 1.39 | -0.37 | -0.45 | 1.63 | -0.32 | -0.07 | 0.66 | -0.05 | 0.01 |
|  | Vegetable intake (times/day) | -0.57 | 1.11 | -0.19 | -0.46 | 1.21 | -0.06 | 0.04 | 0.68 | 0.05 | 0.05 |
|  | Sweet discretionary food intake (times/day) | 0.27 | -0.25 | -0.05 | -0.02 | -0.17 | 0.14 | 0.11 | -0.06 | 0.46 | 0.04 |
|  | Savoury discretionary food intake (times/day) | 0.09 | -0.26 | 0.07 | -0.16 | -0.09 | 0.28 | 0.20 | 0.04 | 0.48 | 0.17 |
| PA | Organised sport (mins/day) | -0.17 | 0.12 | 0.05 | -0.08 | 0.03 | 0.09 | 0.03 | 0.05 | 0.00 | -0.52 |
|  | Outdoor play (mins/day) | -0.39 | 0.39 | 0.06 | -0.28 | 0.30 | 0.24 | 0.16 | 0.28 | -0.03 | -0.33 |
|  | MVPA (mins/day) | -0.82 | -0.17 | 0.64 | -0.60 | -0.13 | 0.95 | 0.65 | 0.00 | -0.01 | -0.05 |
| SB | Screen time (mins/day) | 0.52 | -0.24 | -0.22 | 0.06 | -0.16 | 0.01 | -0.07 | 0.01 | 0.53 | 0.04 |
|  | Videogame time (mins/day) | 0.40 | -0.08 | -0.23 | 0.06 | 0.02 | -0.09 | -0.15 | 0.02 | 0.48 | -0.20 |
|  | Quiet play time (mins/day) | 0.13 | 0.11 | -0.14 | 0.05 | 0.05 | -0.10 | -0.05 | 0.11 | 0.04 | 0.65 |
|  | Sedentary time (mins/day) | 0.75 | 0.14 | -0.58 | 0.59 | 0.12 | -0.93 | -0.65 | 0.02 | -0.02 | 0.01 |
| Sleep | Sleep (mins/day) | -0.42 | -0.04 | 0.30 | -0.10 | 0.06 | 0.11 | 0.21 | 0.02 | -0.19 | 0.34 |

Abbreviations: CA – cluster analysis, LPA – latent profile analysis, mins – minutes, MVPA – moderate- to vigorous-intensity physical activity, PA – physical activity, PCA – principal component analysis, SB – sedentary behaviour.
